# Supplementary material for: Translational control of breast cancer plasticity
Source: Nat Commun. 2020 May 19;11:2498. doi: 10.1038/s41467-020-16352-z (PMC7237473; doi:10.1038/s41467-020-16352-z)
Supplement: Supplementary file 1 — Supplementary Information [file 41467_2020_16352_MOESM1_ESM.pdf]

Translational control of breast cancer plasticity

Jewer et al

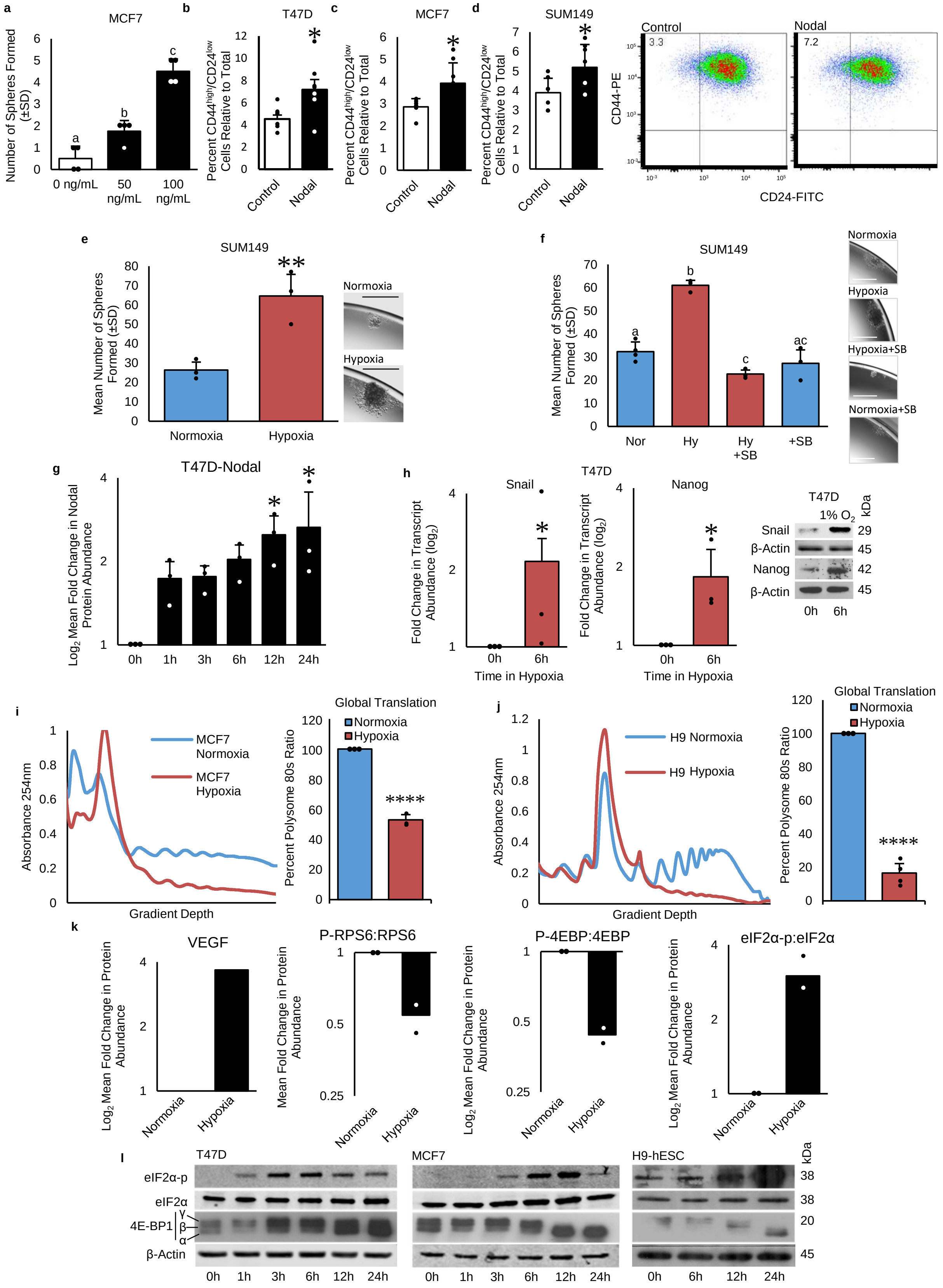
a
MCF7
Number of Spheres Formed (±SD)
0 ng/mL
50 ng/mL
100 ng/mL
b
T47D
Percent CD44high/CD24low Cells Relative to Total
Control
Nodal
c
MCF7
d
SUM149
Control
Nodal
CD44-PE
CD24-FITC
3.3
7.2
e
SUM149
Mean Number of Spheres Formed (±SD)
Normoxia
Hypoxia
f
SUM149
Nor
Hy
Hy +SB
+SB
Hypoxia+SB
Normoxia+SB
g
T47D-Nodal
Log2 Mean Fold Change in Nodal Protein Abundance
0h
1h
3h
6h
12h
24h
h
T47D
Snail
Nanog
Fold Change in Transcript Abundance (log2)
Time in Hypoxia
1% O2
kDa
β-Actin
i
MCF7 Normoxia
MCF7 Hypoxia
Absorbance 254nm
Gradient Depth
Global Translation
Percent Polysome 80s Ratio
j
H9 Normoxia
H9 Hypoxia
k
VEGF
P-RPS6:RPS6
P-4EBP:4EBP
eIF2α-p:eIF2α
Log2 Mean Fold Change in Protein Abundance
Mean Fold Change in Protein Abundance
l
T47D
MCF7
H9-hESC
eIF2α-p
eIF2α
4E-BP1
β-Actin

Supplementary Figure 1: Hypoxia induces BCSC phenotypes and translational reprogramming: a) Mean sphere counts from rhNODAL (50, 100 ng/mL) treated MCF7 cells (n=4). b-d) Mean percentage of CD44high/CD24low population of b) T47D (n=7) c) MCF7 (n=6) and d) SUM149 (n=7) following 24h rhNODAL (100ng/mL) treatement with representative scatterplots defining CD44 and CD24 subpopulations . e) Mean sphere counts from SUM149 cells pre-exposed to hypoxia or normoxia for 24h (n=3) with representative images. Micron bars = 500 µm. f) Mean sphere counts from SUM149 cells pre-exposed to hypoxia or normoxia for 24h +/- SB431542 (10 µM). (n=3) with representative images. Micron bars = 250 µm. g) Mean of the densitometric analysis of Immunoblot of lysates from T47D cells treated for 0-24h in hypoxia (Fig. 1i) (n=3). h) Mean NANOG and SNAIL transcript (qRT-PCR) and protein levels (Immunoblots) in T47D cells cultured in hypoxia for 0 and 6h (n=3). Mean log2 fold change in transcript relative to 0h and β-Actin loading control. Translation rates in i) MCF7 (n=3) and j) H9 (n=4) cells cultured in hypoxia or normoxia for 24h. Bars represent fold change of mRNA associated with polysomes (more than 3 ribosomes) in cells cultured in hypoxia versus normoxia for representative polysome profiles shown. k) Densitometric analysis of VEGF, P-RPS6 relative to RPS6, P-4EBP relative to 4EBP, and eIF2α-P relative to eIF2α (Fig. 1n) (n=2). l) Immunoblots of lysates from T47D breast cancer cells, MCF7 breast cancer cells and H9 hESCs exposed to 20 (normoxia) or 1% O2 (hypoxia) for 0-24h show that low O2 reduces 4E-BP phosphorylation (indicated by a increase in the α and β bands and a relative decrease in the γ band) by 12h, and increases eIF2α phosphorylation between 3 and 12h. 4E-BP1, β-Actin and eIF2α levels were unchanged (n=3). Hypoxia denoted in red and normoxia blue. Data represents independent experiments. Error bars indicate mean ± SD. Two-sided t-test for paired samples. The asterisks denote p-values < *0.05, **0.01, ****0.0001. Multiple comparisons tested by ANOVA. The same letters indicate relationships with a p≥0.05. Different letters indicate statistical differences (p<0.05).

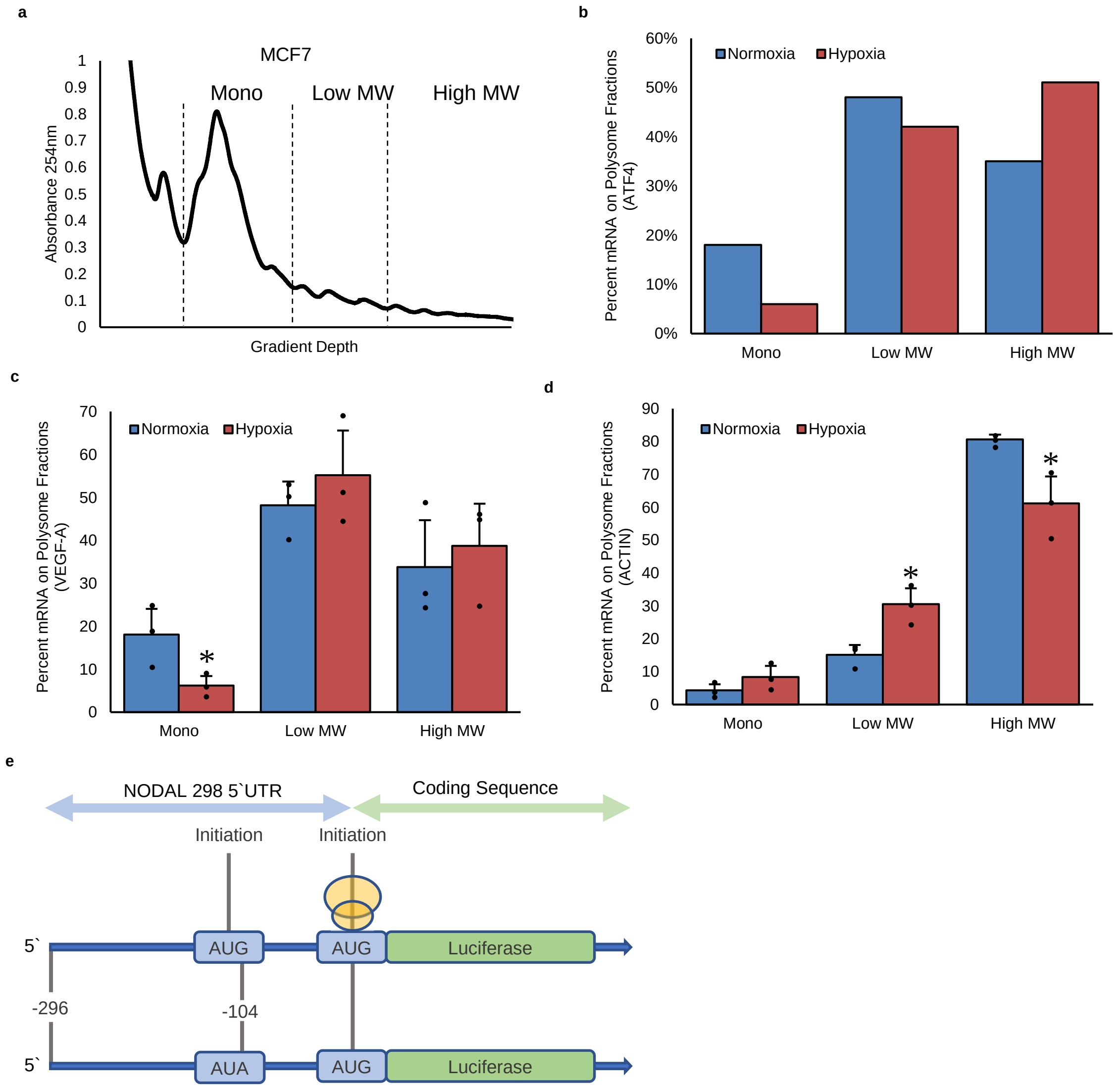

Supplementary Figure 2: Hypoxia induces the selective translation of BCSC-associated transcripts in an isoform-specific manner. a) Representative polysome profile from MCF7 demonstrating the fractions from which monosomes, Low MW polysomes (<4) and High MW polysomes (>4) were taken. b) Percent of ATF4 mRNA associated with mononsomes, low MW polysomes and high MW polysomes extracted from MCF7 cells cultured for 24h in hypoxia or normoxia. Bars represent the percent of transcript associated with each fraction in each condition. A representative biological replicate is shown (n=1). c) Mean percentage of VEGF (n=3) and d) β-ACTIN (n=4) mRNA associated with mononsomes, low MW polysomes and high MW polysomes extracted from MCF7 cells cultured for 24h in hypoxia or normoxia. Bars represent the percent of transcript associated with each fraction in each condition ± SD (n=3). e) Sequence diagram of the NODAL 298 nt 5'UTR highlighting the uORF. Hypoxia denoted in red and normoxia blue. Data represents independent experiments. Error bars indicate mean ± SD. Two-sided t-test for paired samples. The asterisks denote p-values < *0.05.

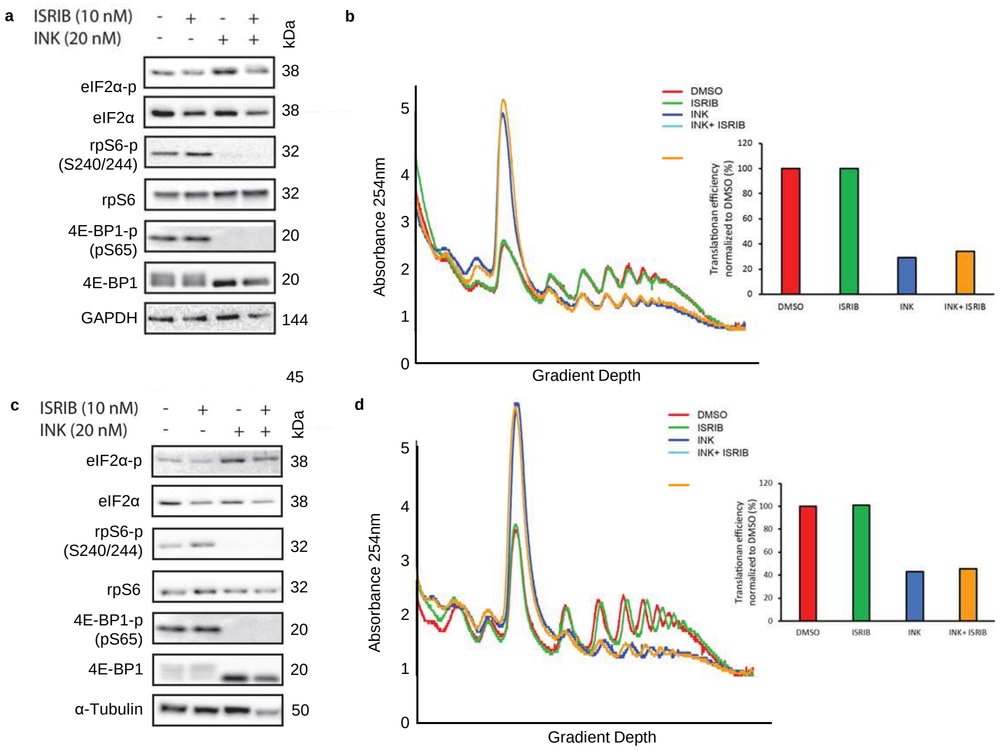

Supplementary Figure 3: mTOR inhibition induces the selective translation of BCSC-associated transcripts in an isoform-specific manner. a) Western blot analysis of key translation regulating proteins (p-EIF2α, 4E-BP1, p-RPS6) in response to ISRIB, INK and INK and ISRIB with respective loading controls (EIF2α, 4E-BP1, RPS6) from T47D lysate. **b)** Second replicate polysome profile from MCF7 demonstrating overall changes in translational efficiency. **c)** Western blot analysis of key translation regulating proteins (p-EIF2α, 4E-BP1, p-RPS6) in response to ISRIB, INK and INK and ISRIB with respective loading controls (EIF2α, 4E-BP1, RPS6) from T47D lysate. **d)** Third replicate polysome profile from MCF7 demonstrating overall changes in translational efficiency.

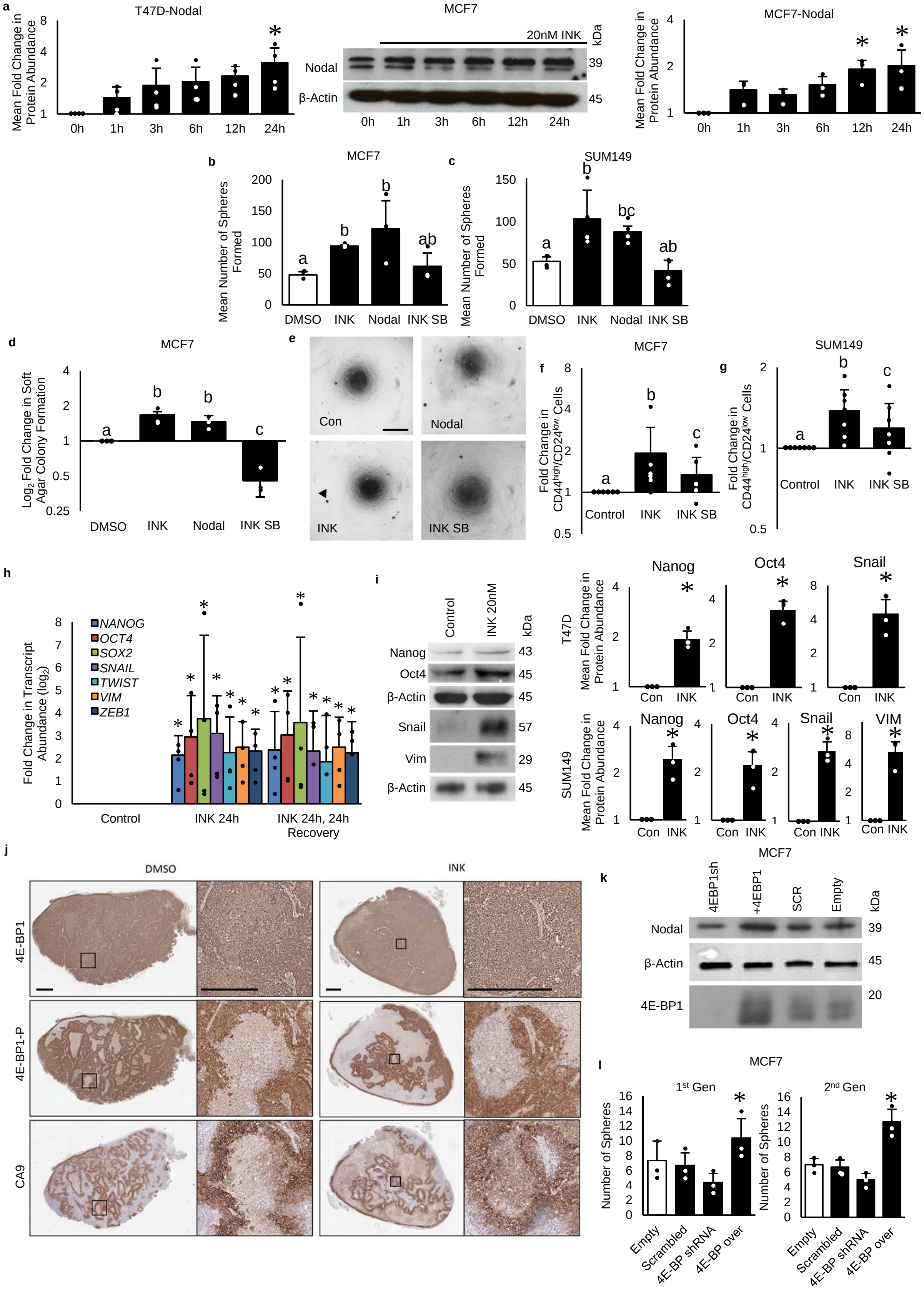

a
T47D-Nodal
Mean Fold Change in Protein Abundance
0h 1h 3h 6h 12h 24h
MCF7
20nM INK
kDa
Nodal 39
β-Actin 45
0h 1h 3h 6h 12h 24h
MCF7-Nodal
Mean Fold Change in Protein Abundance
0h 1h 3h 6h 12h 24h
b
MCF7
Mean Number of Spheres Formed
DMSO INK Nodal INK SB
c
SUM149
Mean Number of Spheres Formed
DMSO INK Nodal INK SB
d
MCF7
Log2 Fold Change in Soft Agar Colony Formation
DMSO INK Nodal INK SB
e
Con
Nodal
INK
INK SB
f
MCF7
Fold Change in CD44high/CD24low Cells
Control INK INK SB
g
SUM149
Fold Change in CD44high/CD24low Cells
Control INK INK SB
h
NANOG
OCT4
SOX2
SNAIL
TWIST
VIM
ZEB1
Fold Change in Transcript Abundance (log2)
Control
INK 24h
INK 24h, 24h Recovery
i
Control
INK 20nM
kDa
Nanog 43
Oct4 45
β-Actin 45
Snail 57
Vim 29
β-Actin 45
T47D
Mean Fold Change in Protein Abundance
Nanog
Oct4
Snail
Con INK
SUM149
Mean Fold Change in Protein Abundance
Nanog
Oct4
Snail
VIM
Con INK
j
DMSO
INK
4E-BP1
4E-BP1-P
CA9
k
MCF7
4EBP1sh
+4EBP1
SCR
Empty
kDa
Nodal 39
β-Actin 45
4E-BP1 20
l
MCF7
1st Gen
2nd Gen
Number of Spheres
Empty
Scrambled
4E-BP shRNA
4E-BP over

Supplementary Figure 4: MTOR inhibition induces breast cancer plasticity: a) Densitometric analysis of NODAL immunoblots from 0-24h 20nM INK treated T47D cells Fig. 4a). NODAL immunoblot and densitometric analysis of 0-24h 20nM INK treated MCF7 cells with β-Actin loading control (n=3). b,c) Mean sphere counts from b) MCF7 and c) SUM149 cells pre-exposed to DMSO, INK (20nM), rhNODAL (100 ng/mL) or INK + SB431542 (10 μM) for 24h (n=4) with representative sphere images. d,e) Mean s colonies formed from d) MCF7 cells pre-exposed to DMSO, INK (20nM), rhNODAL (100 ng/mL) or INK + SB431542 (10 μM) for 24h with representative images. (n=3). Micron bars = 1000 μm f,g) Mean percentage of CD44high/CD24low f) MCF7 (n=7) and g) SUM149 (n=6) cells following DMSO, INK, INK+SB431542 or INK with 24h washed out treatment. h) Mean log2 fold change relative to control qRT-PCR quantification of NANOG, SOX2, OCT4, TWIST, ZEB1, SNAIL, VIM transcripts from 24h treated DMSO or INK (20 nM) SUM149 cells (n=4). i) Relative mean protein expression by immunoblot and densitometric analyses of NANOG, OCT4, SNAIL, VIM in SUM149 cells cultured for 24h in DMSO or INK (20 nM) with β-Actin loading control (n=3). Mean protein expression by densitometric analysis of immunoblots from (Fig. 4f) 24h DMSO or INK (20 nM) treated T47D cells (n=3). j) Immunohistochemical detection (brown) of phospo-4E-BP1, total 4E-BP1 and CA9 in PDX 401 tumours taken from mice treated every second day for 2 weeks with either DMSO or INK (30 mg/kg). Micron bars = 1000 μm and 250 μm. k) Immunoblot fpr NODAL and 4E-BP1 in MCF7 cells stably transfected with 4E-BP1 shRNA, a shRNA scrambled (SCR) control, a 4E-BP1 ORF or an empty vector with β-Actin loading control. l) Mean first and second generation spheres formed from MCF7 cells transfected with vectors as described in k) (n=3) with representative images. Data represents independent experiments. Error bars indicate mean ± SD. Two-sided t-test for paired samples. The asterisks denote p-values < *0.05. Multiple comparisons tested by ANOVA. The same letters indicate relationships with a p≥0.05. Different letters indicate statistical differences (p<0.05).

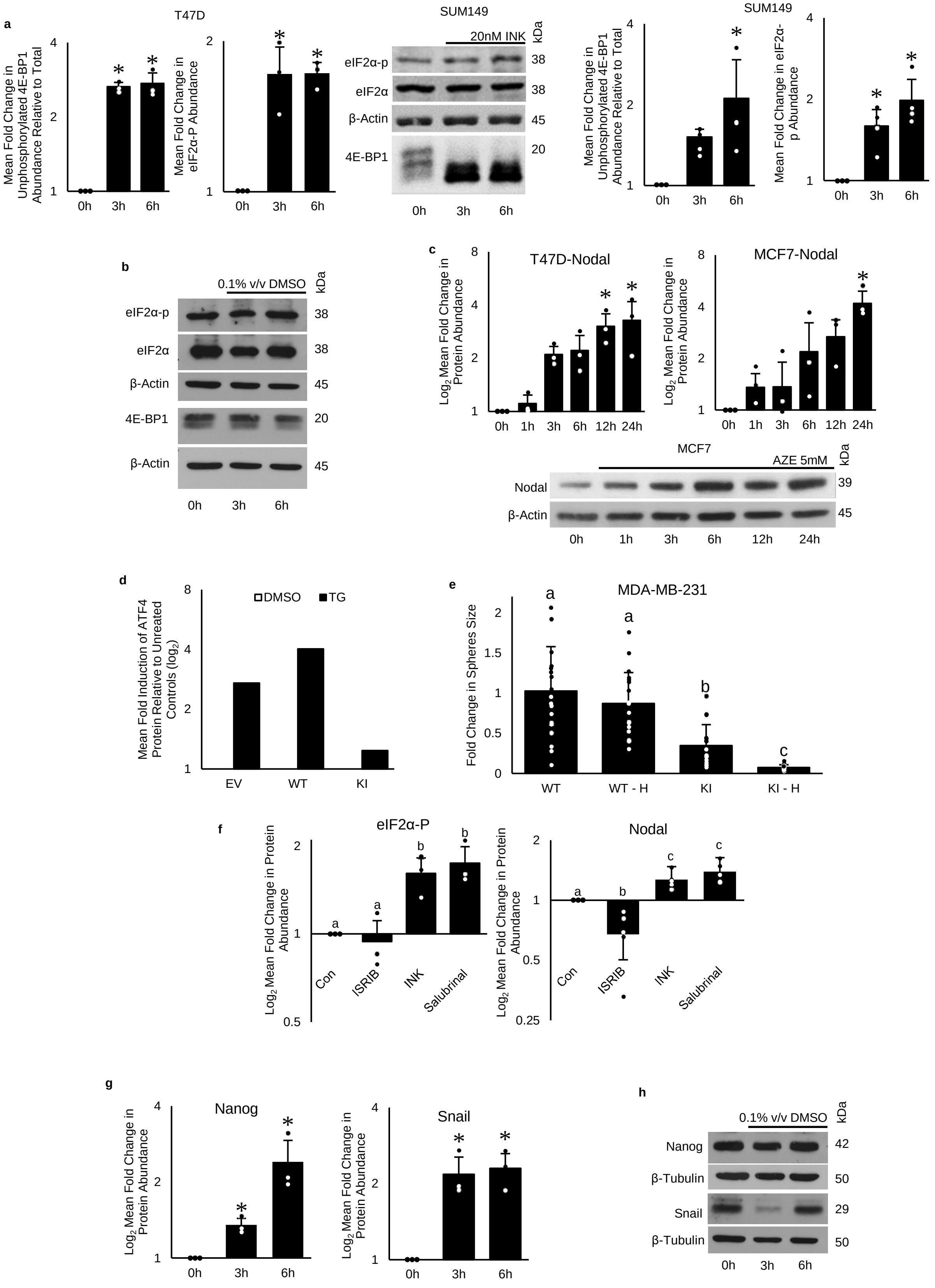
a
T47D
Mean Fold Change in Unphosphorylated 4E-BP1 Abundance Relative to Total
Mean Fold Change in eIF2α-P Abundance
0h
3h
6h
*
SUM149
20nM INK
kDa
eIF2α-p
eIF2α
β-Actin
4E-BP1
38
38
45
20
SUM149
Mean Fold Change in Unphosphorylated 4E-BP1 Abundance Relative to Total
Mean Fold Change in eIF2α-p Abundance
b
0.1% v/v DMSO
kDa
eIF2α-p
eIF2α
β-Actin
4E-BP1
β-Actin
38
38
45
20
45
c
T47D-Nodal
MCF7-Nodal
Log_2 Mean Fold Change in Protein Abundance
0h
1h
3h
6h
12h
24h
MCF7
AZE 5mM
kDa
Nodal
β-Actin
39
45
d
Mean Fold Induction of ATF4 Protein Relative to Unreated Controls (log_2)
DMSO
TG
EV
WT
KI
e
MDA-MB-231
Fold Change in Spheres Size
a
a
b
c
WT
WT - H
KI
KI - H
f
eIF2α-P
Nodal
Log_2 Mean Fold Change in Protein Abundance
Con
ISRIB
INK
Salubrinal
g
Nanog
Snail
Log_2 Mean Fold Change in Protein Abundance
h
0.1% v/v DMSO
kDa
Nanog
β-Tubulin
Snail
β-Tubulin
42
50
29
50

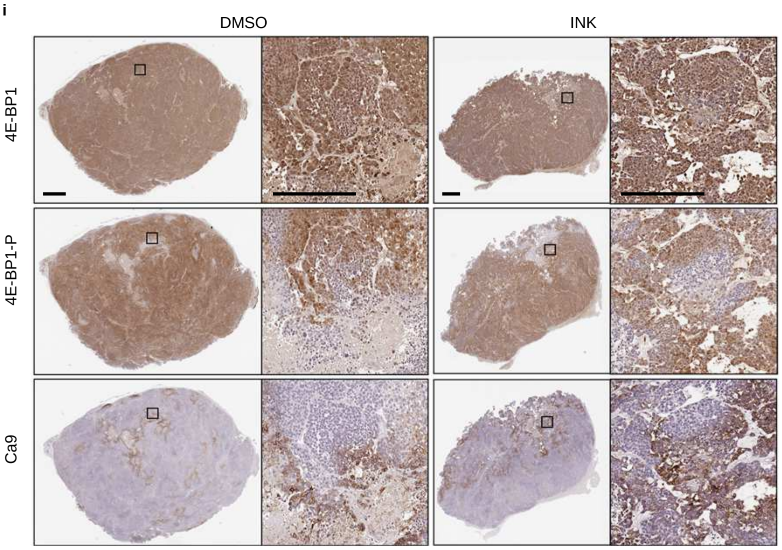
i
DMSO
INK
4E-BP1
4E-BP1-P
Ca9

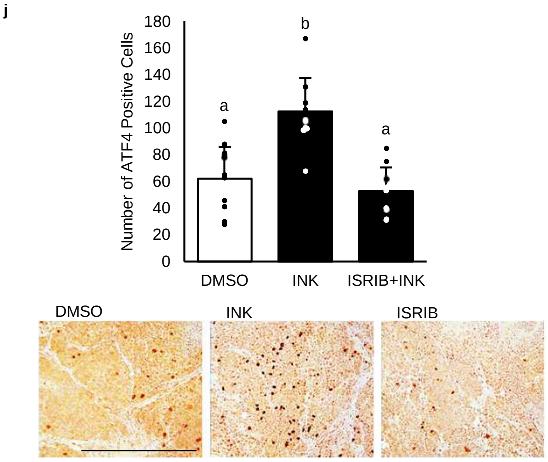
j
Number of ATF4 Positive Cells
180
160
140
120
100
80
60
40
20
0
a
b
a
DMSO
INK
ISRIB+INK
DMSO
INK
ISRIB

Supplementary Figure 5: Stress-induced plasticity is mediated by the ISR: a) Mean densitometric analysis of protein expression by immunoblots (0, 3 or 6h with INK 20 nM treated T47D cells (Fig. 5a)). Mean protein expression of eIF2α-p, eIF2α and 4E-BP1 from 0, 3 or 6h with INK 20 nM treated SUM149 cells (n=4). b) eIF2α-p, eIF2α and 4E-BP1 immunoblot analysis of T47D cells treated for 0, 3 or 6h with 0.1% v/v DMSO solvent. c) Mean protein expression by densitometric analysis of NODAL immunoblot from 0-24h AZE (5mM) treated T47D cells (n=3). d) Densitometric analysis of for mean protein expression of ATF4 immunoblots from MDA-MB-231 cells (vehicle or TG) expressing an empty vector (EV), a wild type eIF2α (WT) or an eIF2 α mutant normalized to untreated expression of ATF4. e) Area of spheres formed by MDA-MB-231 variants described in (Fig. 5c), 24h pre-exposure to hypoxia or normoxia (Fig. 5e) (n=3). f) Densitometric analysis of eIF2α-p and eIF2α immunoblots from 24h vehicle, ISRIB (10nM), INK 20 nM or Salubrinal (20μM) treated T47D cells (Fig. h) (n=3). g) Densitometric analysis of NANOG and SNAIL immunoblots in 0,3 or 6h with Salubrinal (20μM) treated T47D cells (Fig. 5g). h) NANOG, and SNAIL immunoblot analysis from 0, 3 or 6h with 0.1% v/v DMSO T47D cells. β-Tubulin was used as a loading control i) Immunohistochemical detection (brown) of phospo-4E-BP1, total 4E-BP1 and CA9 in SUM149 tumours from mice treated every second day for 2 weeks with DMSO or INK (30 mg/kg). Micron bars = 500 μm. j) Immunohistochemical detection (brown) of ATF4 in SUM149 tumours from mice treated every second day for 2 weeks with DMSO (n=12), INK (30 mg/kg) (n=9) or INK+ISRIB (2.5 mg/kg) (n=3). Micron bars = 400 μm. Data represents independent experiments. Error bars indicate mean ± SD. Two-sided t-test for paired samples. The asterisks denote p-values < *0.05 (c) and g) ANOVA vs control).Multiple comparisons tested by ANOVA. The same letters indicate relationships with a p≥0.05. Different letters indicate statistical differences (p<0.05).

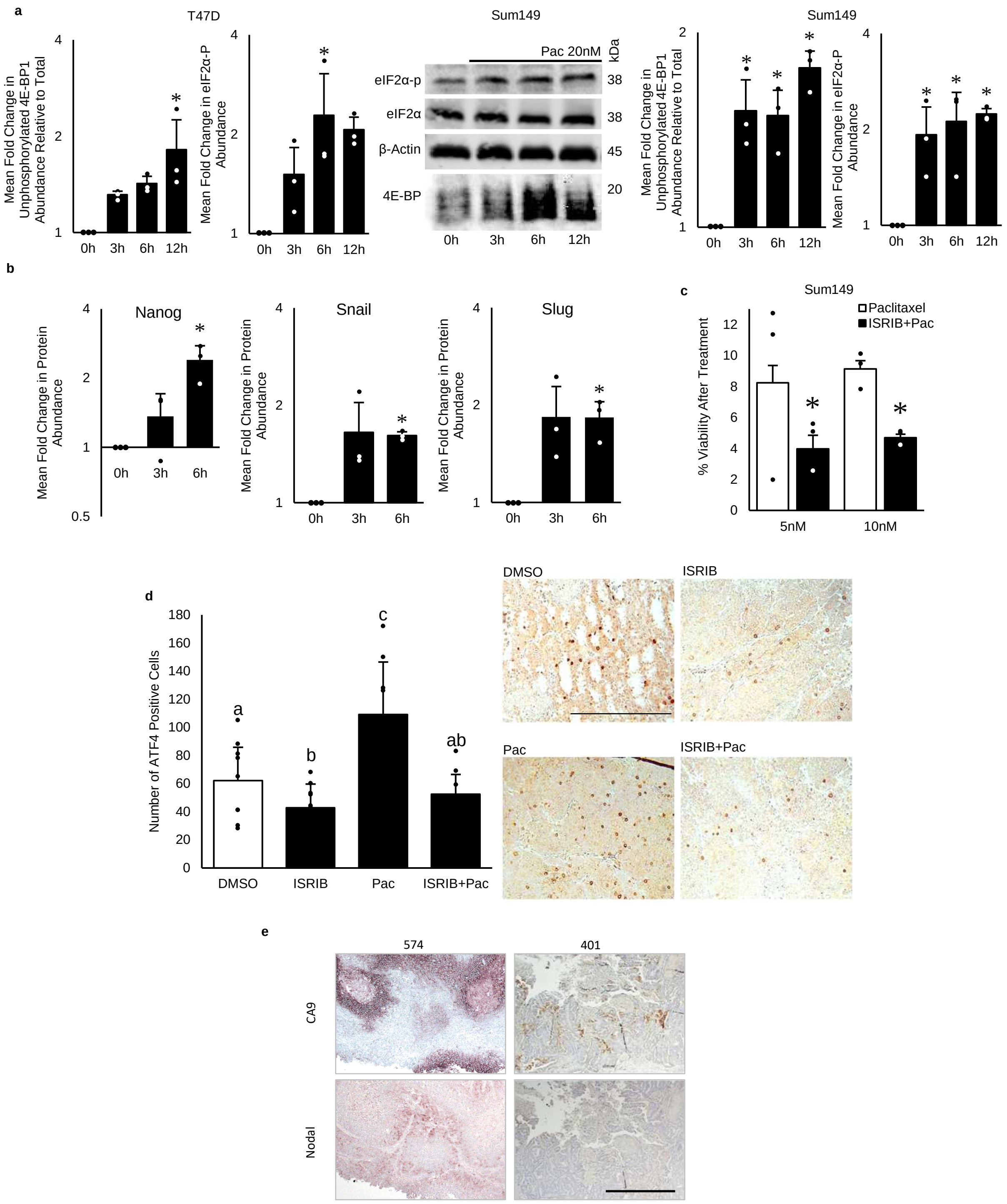
a
T47D
Mean Fold Change in Unphosphorylated 4E-BP1 Abundance Relative to Total
Mean Fold Change in eIF2α-P Abundance
0h
3h
6h
12h
Sum149
Pac 20nM
kDa
eIF2α-p
eIF2α
β-Actin
4E-BP
38
38
45
20
b
Nanog
Snail
Slug
Mean Fold Change in Protein Abundance
c
Sum149
Paclitaxel
ISRIB+Pac
% Viability After Treatment
5nM
10nM
d
Number of ATF4 Positive Cells
DMSO
ISRIB
Pac
ISRIB+Pac
a
b
c
ab
e
574
401
CA9
Nodal

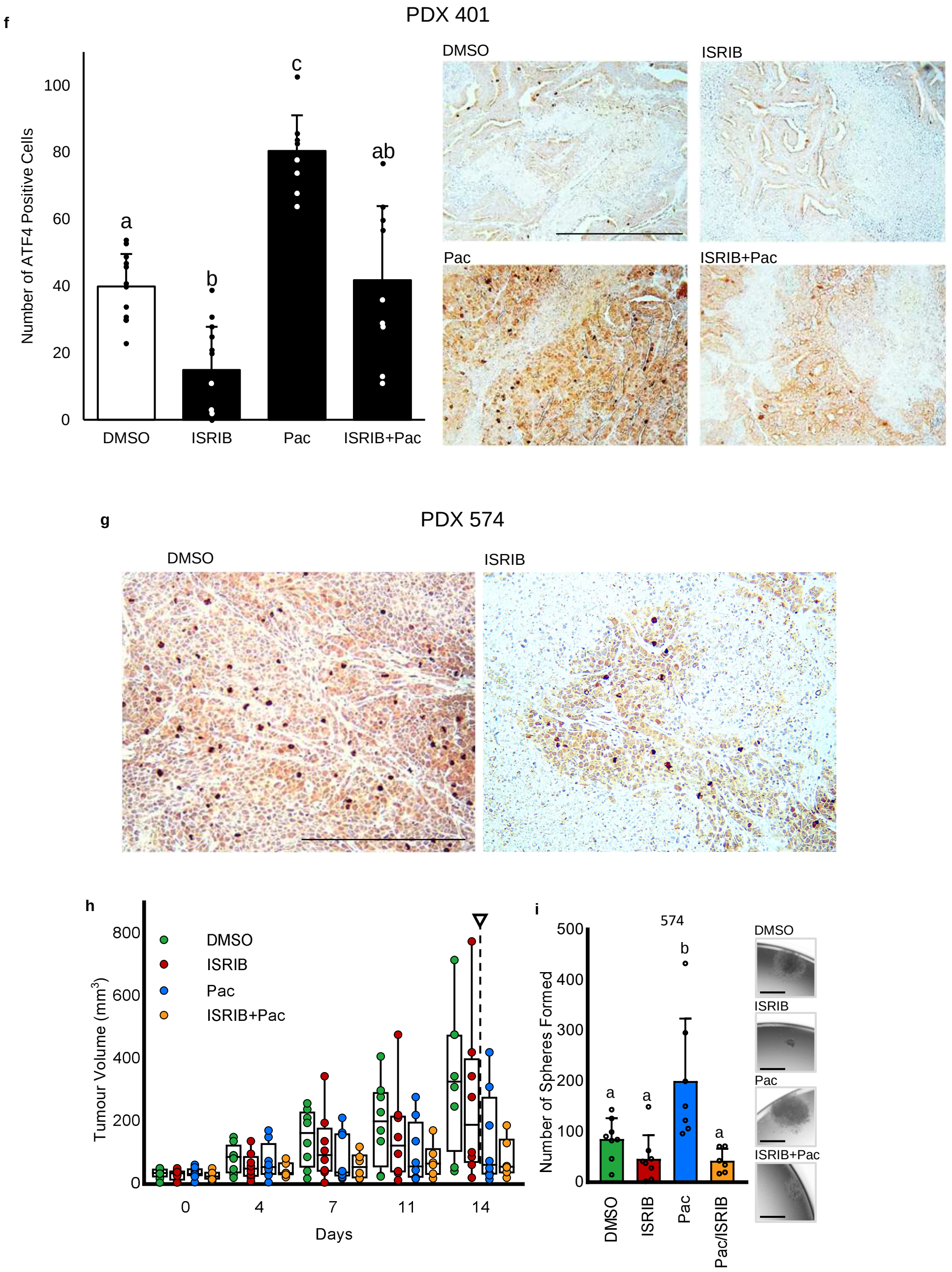
f
PDX 401
Number of ATF4 Positive Cells
0
20
40
60
80
100
a
b
c
ab
DMSO
ISRIB
Pac
ISRIB+Pac
DMSO
ISRIB
Pac
ISRIB+Pac
g
PDX 574
DMSO
ISRIB
h
Tumour Volume (mm3)
0
200
400
600
800
DMSO
ISRIB
Pac
ISRIB+Pac
0
4
7
11
14
Days
i
574
Number of Spheres Formed
0
100
200
300
400
500
a
a
b
a
DMSO
ISRIB
Pac
Pac/ISRIB
DMSO
ISRIB
Pac
ISRIB+Pac

Supplementary Figure 6: ISRIB mitigates therapy-induced BCSCs and improves efficacy of paclitaxel: a) Densitometric analysis of mean protein expression of eIF2α-p, eIF2α and 4E-BP1 immunoblots from 0-12h with paclitaxel (20nM) treated T47D cells (Fig 6a). eIF2α-p, eIF2α and 4E-BP1 immunoblots and densitometric analysis of 0-12h with paclitaxel (20nM) treated SUM149 cells. β-Actin loading control (n=3). b) Mean protein expression (densitometry) of NANOG (n=4) and SNAIL (n=4), SLUG (n=3) immunoblots from 0,3 or 6h paclitaxel treated T47D cells (20nM) (Fig. 6b). c) Mean colony formation of the surviving fractions of 5 nM and 10nM paclitaxel treated SUM149 cells ± ISRIB (10nM) ($p<0.05$, n=3). d) Immunohistochemical detection (brown) of ATF4 in SUM149 tumours from mice treated every second day for 2 weeks with DMSO, ISRIB (2.5 mg/kg), paclitaxel (20 mg/kg), or paclitaxel+ISRIB (2.5 mg/kg). Mean number of ATF+ cells per field of view (n=3). Micron bars = 400 μm. e) Immunohistochemical analysis of CA9 and NODAL (brown) PDX lines 401 and 574. Micron bars = 500 μm. f,g) Immunohistochemical detection (brown) of ATF4 in f) the mean number of ATF+ cells per field of view in PDX401 tumours from mice treated as in d) ($p<0.01$, n=3) with representative images. Micron bars = 400 μm. g) Representative images of DMSO, ISRIB (2.5 mg/kg) treated 574 PDX tumor h) PDX574 tumour volumes in mice treated every second day for 2 weeks with DMSO, ISRIB (10 mg/kg oral), paclitaxel (20 mg/kg), or paclitaxel (20 mg/kg)+ISRIB (10 mg/kg oral). Arrows indicate treatment cessation (n=8). Micron bars = 400 μm i) Mean sphere count from 96000 PDX574 cells dissociated from endpoint tumours from h) (n=6) with representative images. Micron bars = 500 μm. Data represents independent experiments. Error bars indicate mean ± SD. Two-sided t-test for paired samples. The asterisks denote p-values < *0.05 (a) and b) ANOVA vs control). Multiple comparisons tested by ANOVA. The same letters indicate relationships with a $p \geq 0.05$. Different letters indicate statistical differences ($p<0.05$). Box and whisker plots represent median, interquartile range (IQR), whiskers extend to maximum and minimum.

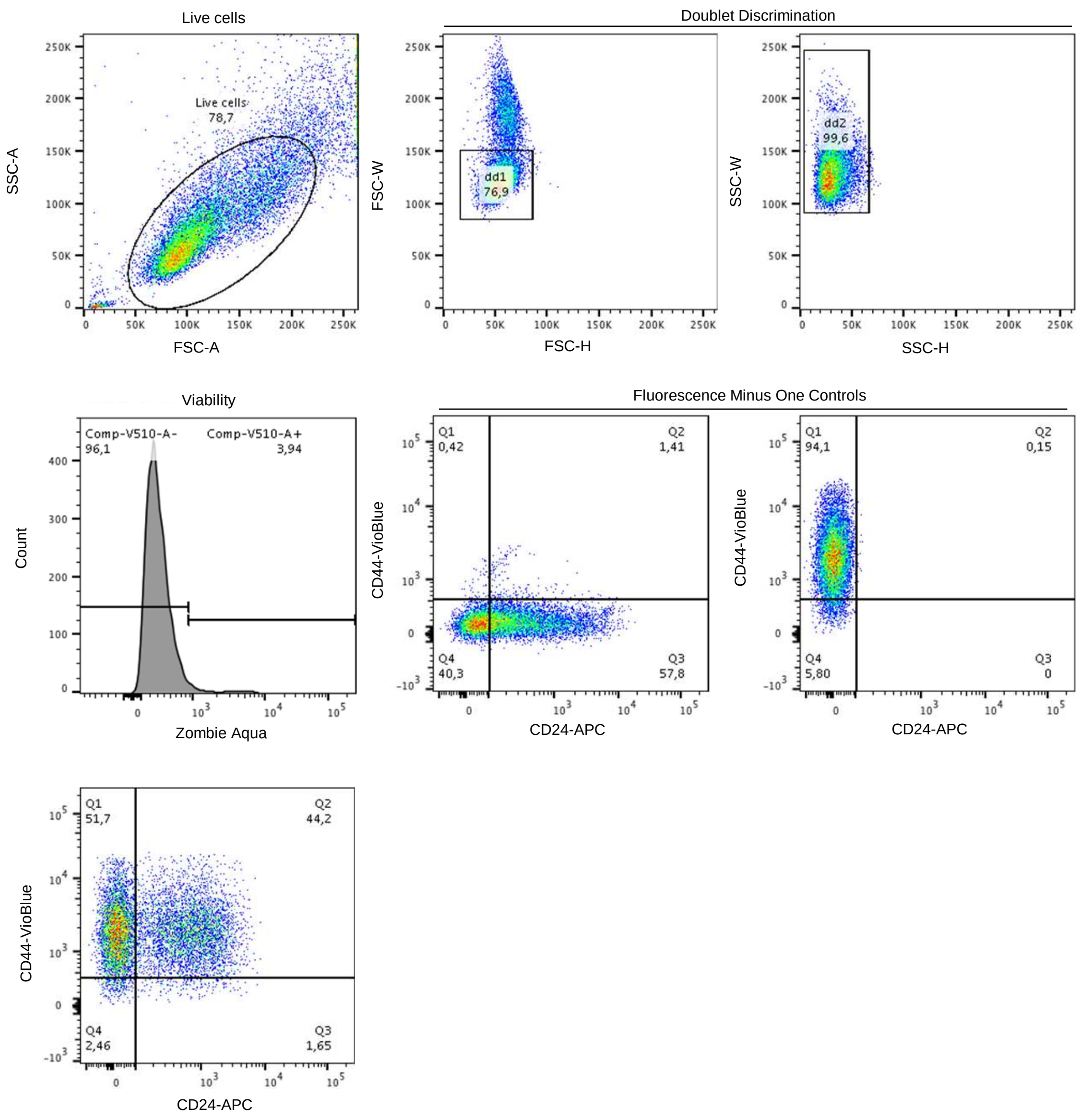

Supplementary Figure 7: Schema of gating as described in the methods: Live cell gating using SSC-A and FSC-A (live cells), doublet discrimination gate (dd1) using FSC-H and FSC-W and SSC-H and SSC-W (dd2). Viable cells, unstained by Zombie Aqua, were selected in gate Com-V510-A-. Fluorescence minus one controls were used to set a quadrant gate to measure DC44High CD24low cells (Q1). Final panel displayed in figures.

Supplementary Table 1. Top 50 Gene Most Altered by Hypoxia in T47D Breast Cancer Cells

genes	logFC	PValue	FDR	genes	logFC	PValue	FDR
LINC01512	9.35074659	1.21E-28	6.32E-27	LINC00867	-4.78129481	4.3956E-31	2.8285E-29
LINC02395	9.00859457	7.06E-27	3.26E-25	LINC02303	-3.89059199	3.5347E-19	7.466E-18
SEMA5B	7.67814516	2.45E-42	3.32E-40	LINC01016	-3.87236614	8.8952E-07	3.6659E-06
FAM131B	7.01146416	4.10E-29	2.19E-27	YEATS2-AS1	-3.49661148	2.0383E-15	2.8953E-14
LOC100506688	6.97537833	1.47E-27	7.07E-26	C1orf64	-3.42521174	8.7727E-23	2.7459E-21
GGTA1P	6.94943554	1.92E-24	7.01E-23	NXPH3	-3.28537879	1.0571E-20	2.6091E-19
BPIFB2	6.84297093	7.83E-27	3.59E-25	KRT13	-3.26754252	3.709E-22	1.058E-20
FGD5	6.66042509	1.40E-109	1.94E-106	PXDNL	-3.21664267	1.2922E-19	2.8639E-18
IGFBP3	6.64408453	8.51E-66	4.06E-63	RANBP3L	-3.03710531	1.8834E-36	1.778E-34
MMP13	6.58612463	2.46E-43	3.55E-41	CXCL14	-2.91694602	0.00332046	0.00673093
HEPHL1	6.47536632	1.49E-34	1.20E-32	KCNK5	-2.90277354	6.7285E-20	1.5336E-18
PPP1R3C	6.46561743	2.53E-21	6.66E-20	PGR	-2.88906536	2.5139E-38	2.5203E-36
MAP7D2	6.29095787	2.27E-87	2.09E-84	ST8SIA1	-2.87260305	3.6078E-09	2.1796E-08
PNMA2	6.23906873	5.15E-73	2.97E-70	DYNC1I1	-2.71305143	4.7474E-15	6.452E-14
TGFBI	6.21467406	3.23E-159	1.12E-155	LRRN3	-2.62060571	5.2721E-14	6.2933E-13
SYT11	6.08408738	2.23E-38	2.25E-36	BCL2	-2.59213144	9.9405E-49	2.183E-46
GABRA2	5.95145268	2.94E-40	3.57E-38	RASL11B	-2.58936217	9.5935E-25	3.6264E-23
SPX	5.92568566	1.16E-26	5.16E-25	SFXN2	-2.55880527	8.4101E-48	1.6388E-45
PTGS1	5.88553584	4.19E-154	1.16E-150	TRPC6	-2.51808971	6.3364E-20	1.4466E-18
MOV10L1	5.87351177	6.43E-40	7.48E-38	MAP6D1	-2.45502009	3.5645E-73	2.1441E-70
BEST1	5.82705475	9.61E-103	1.02E-99	SUSD3	-2.44921078	1.9115E-37	1.8494E-35
NBPF18P	5.82306978	4.39E-26	1.89E-24	LINC01695	-2.4377088	5.6537E-08	2.8526E-07
EPHA3	5.81042545	8.32E-35	6.85E-33	MARC1	-2.42498221	1.026E-47	1.9714E-45
CYP26A1	5.75859213	6.48E-111	9.96E-108	PLIN4	-2.36323604	4.5469E-17	7.6622E-16
STRA6	5.75764828	1.89E-36	1.78E-34	CTPS1	-2.34479867	3.658E-22	1.0456E-20
SLCO2A1	5.71992024	7.26E-47	1.29E-44	ELOVL2	-2.33036229	5.5155E-05	0.00016298
GAD1	5.71312983	5.74E-35	4.82E-33	PDZK1	-2.3287898	9.8651E-05	0.00027791
RORA	5.58903112	1.34E-27	6.46E-26	PTGES	-2.29552548	3.9177E-25	1.5355E-23
SPRR1A	5.56936701	2.24E-31	1.46E-29	CAND2	-2.28306224	3.5501E-12	3.3254E-11
EGLN3	5.53310809	1.10E-43	1.63E-41	OLFML3	-2.23614246	1.7887E-54	5.755E-52
LOX	5.49203174	3.83E-109	4.82E-106	SOWAHA	-2.23381915	9.9952E-21	2.4871E-19
TMEM45A	5.46159251	5.77E-136	1.33E-132	PGR-AS1	-2.22576912	6.4289E-14	7.5633E-13
HPCAL4	5.41296668	1.87E-30	1.12E-28	NDST4	-2.17342335	6.5468E-11	5.1581E-10
SLC28A1	5.38870944	3.95E-32	2.68E-30	CAB39L	-2.16908439	3.0361E-21	7.9403E-20
OPRK1	5.37918209	6.94E-22	1.93E-20	SYN1	-2.15992049	0.00020624	0.00054411
CA9	5.34052635	1.20E-39	1.35E-37	CLPSL1	-2.14690968	1.4361E-25	5.8607E-24
TMPRSS11E	5.23354861	1.60E-28	8.27E-27	PHACTR1	-2.14503016	9.1154E-24	3.0834E-22
INHBA	5.20708476	1.69E-48	3.65E-46	GRIK4	-2.14363726	3.8629E-22	1.0997E-20
LOC105370526	5.18716145	5.35E-59	2.11E-56	PLA2G4F	-2.14129025	1.3652E-21	3.6962E-20
SCN5A	5.16350964	1.48E-38	1.53E-36	GABRG2	-2.13319985	2.4836E-14	3.0844E-13
WNT11	5.16003374	1.55E-24	5.78E-23	SIPA1	-2.1321608	2.6112E-45	4.3007E-43
BGN	5.1179712	3.77E-47	6.86E-45	KCNH1	-2.11852948	1.9713E-25	7.998E-24
HEY1	5.11669441	4.56E-51	1.24E-48	GSTM3	-2.1125358	4.924E-64	2.1975E-61
RASSF10	5.0750622	4.49E-30	2.62E-28	RBM24	-2.0991275	1.55E-76	1.0212E-73
NGFR	5.05728636	2.28E-33	1.73E-31	PGP	-2.06515789	1.4602E-12	1.4348E-11
ATOH8	5.02599427	1.16E-30	7.15E-29	ARHGAP15	-2.06007209	6.5484E-16	9.7942E-15
LOC100128076	4.96691914	6.65E-22	1.86E-20	LINC00565	-2.05971214	2.1392E-11	1.8146E-10
SERPINE1	4.95740356	5.65E-10	3.87E-09	TMEM52B	-1.98960074	1.602E-35	1.394E-33
INHBE	4.94966989	1.68E-49	4.00E-47	PART1	-1.9685209	1.0912E-07	5.2641E-07
CAND1.11	4.94632674	1.88E-28	9.58E-27	GPD1	-1.93696686	9.301E-10	6.1422E-09

Supplementary Table 2. Antibodies Used

Target	Cat #	Protocol	Dilution	Source	Validation
4E-BP1	9644	WB/IHC	1:1000/1:1200	Cell Signaling	4E-BP1 (53H11) Rabbit mAb<br><br>Western blot validation has been done in multiple cells lines (CF-7 HepG2 HeLa 293 PANC1 RD A204 SH-SY5Y), IHC was performed with a blocking peptide control, and specificity was verified in knockout animal models.<br><br>Species Reactivity: Human, Mouse, Rat, Monkey<br><br>Product citations: 1102
β-Actin	sc47778	WB	1:1000	Santa Cruz	β-Actin Antibody (C4) Mouse mAb<br><br>Western blot use has been verified in multiple cell lines.<br><br>Product citations: <5000
ATF4	11815	WB	1:1000	Cell Signaling	ATF-4 (D4B8) Rabbit mAb<br><br>Validation was performed using ER stress induction by tunicamycin in two different cell lines (293 and HeLa cells).<br><br>Product citations: 115
ATF4	ab31390	IHC	1:1000	Abcam	ATF4 ab31390 Rabbit pAb<br><br>Validated by staining ATF-4 in human breast carcinoma tissue compared against the same tissue in the presence of a blocking peptide.<br><br>Reacts with: Mouse, Human
CA9	5648	IHC	1:1000	Cell Signaling	CA9 (D10C10) Rabbit mAb<br><br>Validated against multiple cell lines (LN18 and SW620), and the human colon where it shows the same localization as in the protein atlas. https://www.proteinatlas.org/ENSG00000107159-CA9/tissue/colon<br><br>Species Reactivity: Human
CA9	ab108351	IHC	1:100	Abcam	CA9 ab108351 Rabbit mAb<br><br>Validated for western blot against HT-29 and human stomach, and for IHC in the human stomach, where it shows the same tissue and cellular localization as in the protein atlas. https://www.proteinatlas.org/ENSG00000107159-CA9/tissue/stomach
CD24	130-095-954	Flow	1:20	MiltenyiBiotec	Recombinant Antibodies<br><br>Manufacture validation on Human peripheral blood cells after erythrocyte lysis. Validated on multiple breast cancer cell lines with know expression of CD24. MDA-MB-231 cells possess both a negative and positive population and T47D cells are primarily CD24 positive. Expression is also tested against a Isotype control.<br><br>Product citations: 2 (APC only)
CD44	550989	Flow	1:5	MiltenyiBiotec	Mouse mAb<br><br>Manufacture validation on Human peripheral blood mononuclear cells .Validated on multiple breast cancer cell lines with know expression of CD44. MDA-MB-231 and SUM149 cells are primarily for CD44 expression. Expression is also tested against an Isotype control.<br><br>Product citations: 7 (Vioblue only)
CD24	555428	Flow	1:5	BDBioscience	Mouse mAb<br><br>Validated on multiple breast cancer cell lines with know expression of CD24. MDA-MB-231 cells possess both a negative and positive population and T47D cells are primarily CD24 positive. Expression is also tested against an Isotype control.<br><br>Product citations: 122 (FITC only)
CD44	550989	Flow	1:5	BDBioscience	Mouse mAb<br><br>Validated on multiple breast cancer cell lines with know expression of CD44. MDA-MB-231 and SUM149 cells are primarily for CD44 expression. Expression is also tested against an Isotype control.<br><br>Product citations: 55 (PE only)
eIF2α	9722	WB	1:1000	Cell Signaling	eIF2α Antibody Rabbit pAb<br><br>Validated for western blot in conjunction with Phospho-eIF2α in response to thapsigargin.<br><br>Product citations: 328
eIF2α-P	9721	WB	1:1000	Cell Signaling	Phospho-eIF2α (Ser51) Antibody Rabbit pAb<br><br>Validated western blot in conjunction with eIF2α in response to thapsigargin.<br><br>Product citations: 382

Supplementary Table 2. Antibodies Used Continued

Target	Cat #	Protocol	Dilution	Source	Validation
Nodal	PA5-28486	IHC	1:1000	ThermoFisher Scientific	Mouse pAb<br>Antibody was validated by pathologist Dr. Martin Koebel, University of Calgary, AB Canada using knockout cell lines and tumour microarrays.<br>Species reactivity: Human<br>Citations: 11
Nodal	sc81953	WB	1:1000	Santa Cruz	Mouse mAb<br>Western blot analysis of Nodal expression in HeLa whole cell lysate and human recombinant Nodal fusion protein<br>Citations: 4
Oct4	2840	WB	1:1000	Cell Signalin	Rabbit mAb<br>Western blot analysis of extracts from NTERA2 and mouse embryonic stem cells (mESCs) using Oct-4A (C30A3). H9, and H1 hESC lysate.<br>Citation: 64
P-4E-BP	2855	IHC	1:1600	Cell Signaling	Rabbit mAb<br>Immunohistochemical analysis of paraffin-embedded human colon carcinoma using Phospho-4E-BP1 (Thr37/46) (236B4) Rabbit mAb.<br>Species reactivity: Human, mouse, rat, monkey, D. melanogaster<br>Citations: 502
phospho-4EBP1 (S65)	9451	WB	1:1000	Cell Signaling	Rabbit pAb<br>Western blot analysis of extracts from 293 cells using 4E-BP1 Antibody #9644 and Phospho-4E-BP1 (Ser65) Antibody #9451. The cells were starved for 24 hours in serum-free medium and underwent a 1 hour amino acid deprivation. Amino acids were replenished for 1 hour. Cells were then either untreated (-) or treated with 100 nM insulin (+) for 30 minutes.<br>Citations: 190
phospho-RPS6 (S240/244)	2215	WB	1:1000	Cell Signaling	Rabbit pAb<br>Western blot analysis of extracts from 293 cells, untreated or treated with 20% FBS for the indicated time, using Phospho-S6 Ribosomal Protein (Ser235/236) Antibody #2211 and Phospho-S6 Ribosomal Protein (Ser240/244) Antibody #2215.<br>Citations: 334
Slug	9585	WB	1:1000	Cell Signaling	Rabbit Monoclonal Antibody<br>Western blot analysis of extracts from A204, SKMEL5, and NIH/3T3 cells using Slug (C19G7) Rabbit mAb.<br>Citations: 203
Snail	3879	WB	1:1000	Cell Signaling	Rabbit mAb<br>Western blot analysis of extracts from HCT116, HeLa, NIH/3T3, Rat2, and COS cells using Snail (C15D3) Rabbit mAb.<br>Citations: 209
Sox	3579	WB	1:1000	Cell Signaling	Rabbit mAb<br>Western blot analysis of extracts from NTERA2 and NCCIT cells using Sox2 (D6D9) XP® Rabbit mAb.<br>Citations: 98
Total RPS6	2217	WB	1:1000	Li-cor	Rabbit mAb<br>Western blot analysis of extracts from HeLa, NIH/3T3, PC12 and COS cells using S6 Ribosomal Protein (5G10) Rabbit mAb.<br>Citations: 683
Twist	ab50581	WB	1:1000	Abcam	Rabbit pAb<br>Twist detected via western blot in 293T WT and 293T-Twist transfected cell lysate. Twist staining in murine brain tissue by immunohistochemistry.<br>Citations: 52
VEGFA	A12303	WB	1:1000	ABclonal	Rabbit pAb<br>Wesern bloting using MCF7, rat brain, mouse and rat kidney, mouse bone tissue, and immunofluorescence in HUVEC cells.<br>Citations: 3
α-Tubulin	926-42213	WB	1:1000	Li-cor	Mouse mAb<br>α-Tubulin detected via western blot in HeLa, NIH/3T3, and COS7 cell lysates using 926-42211 and IRDye 680LT Goat Anti-Mouse.
β-Actin	4970	WB	1:1000	Cell Signaling	Rabbit mAb<br>Western blot analysis of cell extracts from NIH/3T3, HeLa, PAE, and A431 cell lysates using beta-Actin (13E5) Rabbit mAb.<br>Product citations: 1102
β-Tubulin	926-42211	WB	1:1000	Cell Signaling	Rabbit pAb<br>β-Tubulin detected via western blot in HeLa, NIH/3T3, and COS7 cell lysates using 926-42211 and IRDye 680 Goat Anti-Rabbit.

Supplementary Table 3. Primer Probes Used

Gene	Cat #	Assay ID
Actin	4331182	HS01060665_g1
ATF4	4331182	Hs00909569_g1
ATF4	12001950	qMmuCEP0056683
EEF2	4331182	Hs00157330_m1
Lox	4331182	Hs00942480_m1
Nanog	4331182	Hs04260366_g1
Nodal	4331182	Hs00415443_m1
Oct4	4331182	HS04260367_g1
RPS8	4331182	Hs04195024_g1
Slug	4331182	HS009500344_g1
Snail (snai1)	4331182	Hs00195591_m1
Sox2	4331182	Hs01053049_s1
Twist	4331182	Hs01675818_s1
Vegf	4331182	Hs00900055_ml
Vimentin (VIM)	4331182	Hs00185584_m1
Zeb1	4331182	Hs00232783_m1

Supplementary Table 4. G Blocks Used in Luciferase Reporter Assays

gBlock Sequences:

>Nanog_350

ATATATATCGTCTCAACACCGAGCGACCCTGCATAAGCTTGACACAATGGGACAGGGAGCGGGGGATGGGGGAATTCAGCTCAGGCTTT
TATGCAAAGACCCCCTTCTGCAAAGAACAAAGCTTCTGGTACCTGCCCTTTGGAGAGCTGCGGGCAAGCTCAGCCTCGAAACACACACA
CCCACACGAGATGGGCACGGAGTAGTCTTGAAAGACATGACAAATCACCAGACCTGGGAAGAAGCTAAAGAGCCAGAGGGAAAAAGCC
AGAAGTCGACTACCTGGGAGGAGGGATAGACAAGAAACCAAACTAAAGGAAACTAAGTGTGGATCCAGCTTGTCCCCAAAGCTTGCCTT
GCTTTGAAGCATCCGACTGTAAAGAATCTTCACCTATGGAAGAGAGACGTATATATA

>Nanog_219

ATATATATCGTCTCAACACCGAGCGACCCTGCATAAGCTTTTCATTATAAATCTAGAGACTCCAGGATTTTAACGTTCTGCTGGACTGAGC
TGGTTGCCTCATGTTATTATGCAGGCAACTCACTTTATCCCAATTTCTTGATACTTTTCCTTCTGGAGGTCCTATTTCTCTAACATCTTCCAG
AAAAGTCTTAAAGCTGCCTTAACCTTTTTTCCAGTCCACCTCTTAAATTTTTTCCTCCTCTTCCTCTATACTAACATGGAAGAGAGACGTATA
TATA

>Snail_85

ATATATATCGTCTCAACACCGAGCGACCCTGCATAAGCTTCATTCATTGCGCCGCGGCACGGCCTAGCGAGTGGTTCTTCTGCGCTACTG
CTGCGCGAATCGGCGACCCCAGTGCCTCGACCACTATGGAAGAGAGACGTATATATA

>NODAL_298

ATATATATCGTCTCAACACCGAGCGACCCTGCATAAGCTTCAGCATCAAGGCGTTTGGTACCTAGAGGAGCAGGGTGCCAGGGTGTCCA
GACCGGGCAAACCAGGAGCCCTAGATCCCGTCCCTGGAACTAGGTCCTCCGCCAGCTTTGCGGGCCTCCTCCGCACCAGGCTCAGGTC
TCGCCCATCCCCAGCAGGAGGTCTCTCTGCCCCCCACCCTCCGGAGGGGGGTTATATGATCTTAAAGCTTCCCCAGAGGGAGGAAAGG
TGGGGGCGGGGCGGCTGCTGAGGCCCAGGATATAAGGGCTGGAGGTGCTGCTTTCAGGCCTGGCCAGCCCACCATGGAAGAGAGACG
TATATATA

>NODAL_42

CCTGGAACTAGGTCCTCCGCCAGCTTTGCGGGCCTCCTCCATATATATCGTCTCAACACCGAGCGACCCTGCATAAGCTTATAAGGGCT
GGAGGTGCTGCTTTCAGGCCTGGCCAGCCCACCATGGAAGAGAGACGTATATATA

>NODAL_14

CCTGGAACTAGGTCCTCCGCCAGCTTTGCGGGCCTCCTCCATATATATCGTCTCAACACCGAGCGACCCTGCATAAGCTTCTGGCCAGC
CCACCATGGAAGAGAGACGTATATATAATGCACGCCCACTGCCTGCCCTTCCTTCTGCACGCCTGGT

>NODAL_ALT

ATATATATCGTCTCAACACCGAGCGACCCTGCATAAGCTTACCAGGCAGGGATTGGGGGATGTGGGCAACAAGGGTAACACCTTTCCTC
CTGAAATCACTGGGGTGATGTCTGCCTGTCGGGGAAAAGCCTGATGAACCAGGATCACCCCGTTGGCTCCATCTGGCTCCAGCTTCTGC
TGAACACTCCCTTTTCAGGTGGAGAAGTCATCGTCCTGGTCCCTCCTGCCTTCTGTCTTCCCCAGGAAAGCACTTCAGTATTTGGTAGGG
CCAGGGGTGCTCCCCGAGGCCATCTAGACAGTGGCCAGGGAGGCTGCGCCAGCCAGATTGTGTCAAAGTCTGCCTGAGAGCCACGAG
GATCCCTCGGCATTCTCTTCCTGCTTCTTGAGGAGCCTGCGGTCCATTTGCAGCCTGAGTGGAGAGGATGGGGCAGGGATCCCAGGCCT
GGCCAGCCCACCATGGAAGAGAGACGTATATATA
