# Supplementary material for: Translational control of breast cancer plasticity
Source: Nat Commun. 2020 May 19;11:2498. doi: 10.1038/s41467-020-16352-z (PMC7237473; doi:10.1038/s41467-020-16352-z)
Supplement: Supplementary file 3 — Reporting summary [file 41467_2020_16352_MOESM3_ESM.pdf]

nature research

Corresponding author(s): Lynne-Marie Postovit

Last updated by author(s): 2020-04-01

Reporting Summary

Nature Research wishes to improve the reproducibility of the work that we publish. This form provides structure for consistency and transparency in reporting. For further information on Nature Research policies, see Authors & Referees and the Editorial Policy Checklist.

Statistics

For all statistical analyses, confirm that the following items are present in the figure legend, table legend, main text, or Methods section.

n/a	Confirmed	
☐	☒	The exact sample size (*n*) for each experimental group/condition, given as a discrete number and unit of measurement
☐	☒	A statement on whether measurements were taken from distinct samples or whether the same sample was measured repeatedly
☐	☒	The statistical test(s) used AND whether they are one- or two-sided<br>*Only common tests should be described solely by name; describe more complex techniques in the Methods section.*
☐	☒	A description of all covariates tested
☐	☒	A description of any assumptions or corrections, such as tests of normality and adjustment for multiple comparisons
☐	☒	A full description of the statistical parameters including central tendency (e.g. means) or other basic estimates (e.g. regression coefficient) AND variation (e.g. standard deviation) or associated estimates of uncertainty (e.g. confidence intervals)
☒	☐	For null hypothesis testing, the test statistic (e.g. *F*, *t*, *r*) with confidence intervals, effect sizes, degrees of freedom and *P* value noted<br>*Give P values as exact values whenever suitable.*
☒	☐	For Bayesian analysis, information on the choice of priors and Markov chain Monte Carlo settings
☒	☐	For hierarchical and complex designs, identification of the appropriate level for tests and full reporting of outcomes
☐	☒	Estimates of effect sizes (e.g. Cohen's *d*, Pearson's *r*), indicating how they were calculated

Our web collection on statistics for biologists contains articles on many of the points above.

Software and code

Policy information about availability of computer code

Data collection	No software was used for data collection.
Data analysis	Bigwig v3.3.0 (from deepTools), DeepTools v3.3.0, FeatureCounts v1.5.1, edgeR v3.28.0, FastQC v0.11.5, GAGE v2.26.3, ggplot2 v2.1.0, limma v3.28.6, plyr v1.8.4, PROC v1.8, Skewer v0.1.127, STAR v2.5.2a, survival (v2.39-5).<br><br>ImageJ 1.51, Graphpad 7.04<br><br>Molecular Signatures Database v6.0.<br>Human Refseq GRCh38/Hg19 Assembly, RNA Subcellular CAGE Localization from ENCODE/RIKEN (Release 4 (July 2012))<br>Clinical and Level 3 TCGA RNAseqV2 gene expression data was obtained from the TCGA Data Portal in April 2015.

For manuscripts utilizing custom algorithms or software that are central to the research but not yet described in published literature, software must be made available to editors/reviewers. We strongly encourage code deposition in a community repository (e.g. GitHub). See the Nature Research guidelines for submitting code & software for further information.

Data

Policy information about availability of data

All manuscripts must include a data availability statement. This statement should provide the following information, where applicable:

- Accession codes, unique identifiers, or web links for publicly available datasets
- A list of figures that have associated raw data
- A description of any restrictions on data availability

The list of top 50 differentially expressed genes is included in the supporting files:
"The data that support the findings of this study are available from the corresponding author upon reasonable request."

Field-specific reporting

Please select the one below that is the best fit for your research. If you are not sure, read the appropriate sections before making your selection.

☒ Life sciences ☐ Behavioural & social sciences ☐ Ecological, evolutionary & environmental sciences

For a reference copy of the document with all sections, see nature.com/documents/nr-reporting-summary-flat.pdf

Life sciences study design

All studies must disclose on these points even when the disclosure is negative.

Sample size	As we could not predict the outcome of the experiments, sample size calculations were not made. We used the smallest number of animals needed to obtain statistical significance.
Data exclusions	No animals were omitted, and all data points are presented in the graphs. Statistics were applied as defined in the methods section and all n values were declared in the manuscript. While we do not remove any data points, we would consider 2 SD deviations away from the mean as an outlier.
Replication	All experiments represent independent samples and all attempts at replication were successful. All experiments were repeated to obtain biological replicates as indicated. In most cases, multiple cell lines or PDX models were used.
Randomization	For animal studies the mice from each breeding pair were randomly assigned to each treatment group.
Blinding	Due to the nature of the experiments, we did not blind researches to the samples. However, all attempts were made to minimize confounding variables. Thusly, all replicates were independent. For all experiments for which statistics are shown, the experiments were completed a minimum of three times. The major of the experiments are replicated in second or third cell lines, often representing different stages of disease to minimize cell type and stage-specific effects. Where possible phenomena are replicated using several stimuli including overexpression and chemical inhibitors or multiple stress inducers. In vivo experiments were performed on a minimum of six tumour samples. For cell line experiments, treatment and control cells were counted and plated. After attachment and recovery, cells were treated from the longest treatment to the shortest and all extracted at the same time to control in culture. Cells were monitored for viability and density and the end of treatment.

Reporting for specific materials, systems and methods

We require information from authors about some types of materials, experimental systems and methods used in many studies. Here, indicate whether each material, system or method listed is relevant to your study. If you are not sure if a list item applies to your research, read the appropriate section before selecting a response.

Materials & experimental systems

n/a	Involved in the study
☐	☒ Antibodies
☐	☒ Eukaryotic cell lines
☒	☐ Palaeontology
☐	☒ Animals and other organisms
☒	☐ Human research participants
☒	☐ Clinical data

Methods

n/a	Involved in the study
☐	☐ ChIP-seq
☐	☒ Flow cytometry
☐	☐ MRI-based neuroimaging

Antibodies

Antibodies used	All the antibodies used in the production of this manuscript are include in Supplementary table 2.
Validation	Descriptions of the antibody validation, species, reactivity are all included in Supplementary table 2.

Eukaryotic cell lines

Policy information about cell lines

Cell line source(s)	MDA-MB-231, T47D and MCF7 cells, obtained from ATCC (Manassas, Virginia, USA). SUM 149 cells, purchased from BioreclamationlVT, H9 cells were purchased from WiCell.
Authentication	The cell lines used in this study were verified by STR profiling at The Centre for Applied Genomics (TCAG) at The Hospital for Sick Children.

Mycoplasma contamination	Takara PCR Mycoplasma Detection Set was used to confirm the cells were free of contamination Mycoplasma.
Commonly misidentified lines (See ICLAC register)	No commonly misidentified cell lines were used in the study.

Animals and other organisms

Policy information about studies involving animals; ARRIVE guidelines recommended for reporting animal research

Laboratory animals	All experiments involving animals were approved by the Animal Use Subcommittee at the University of Alberta (AUP00001288 and AUP00001685). The animals used were female Nod scid il2r gamma null age: 6-12 weeks supplied by Jackson Laboratories. Mice were housed at 22C at a humidity of 40-60% in microisolator with pressurized individually ventilated cages with a 12 hour dark light cycle.
Wild animals	No wild animals were used in the study.
Field-collected samples	No field collected samples were used in the study.
Ethics oversight	All experiments involving animals were approved by the Animal Use Subcommittee at the University of Alberta (AUP00001288 and AUP00001685).

Note that full information on the approval of the study protocol must also be provided in the manuscript.

ChIP-seq

Data deposition

- [ ] Confirm that both raw and final processed data have been deposited in a public database such as GEO.
- [ ] Confirm that you have deposited or provided access to graph files (e.g. BED files) for the called peaks.

Data access links<br>*May remain private before publication.*	*For "Initial submission" or "Revised version" documents, provide reviewer access links. For your "Final submission" document, provide a link to the deposited data.*
Files in database submission	*Provide a list of all files available in the database submission.*
Genome browser session (e.g. UCSC)	*Provide a link to an anonymized genome browser session for "Initial submission" and "Revised version" documents only, to enable peer review. Write "no longer applicable" for "Final submission" documents.*

Methodology

Replicates	*Describe the experimental replicates, specifying number, type and replicate agreement.*
Sequencing depth	*Describe the sequencing depth for each experiment, providing the total number of reads, uniquely mapped reads, length of reads and whether they were paired- or single-end.*
Antibodies	*Describe the antibodies used for the ChIP-seq experiments; as applicable, provide supplier name, catalog number, clone name, and lot number.*
Peak calling parameters	*Specify the command line program and parameters used for read mapping and peak calling, including the ChIP, control and index files used.*
Data quality	*Describe the methods used to ensure data quality in full detail, including how many peaks are at FDR 5% and above 5-fold enrichment.*
Software	*Describe the software used to collect and analyze the ChIP-seq data. For custom code that has been deposited into a community repository, provide accession details.*

Flow Cytometry

Plots

Confirm that:

- [x] The axis labels state the marker and fluorochrome used (e.g. CD4-FITC).
- [x] The axis scales are clearly visible. Include numbers along axes only for bottom left plot of group (a 'group' is an analysis of identical markers).
- [x] All plots are contour plots with outliers or pseudocolor plots.
- [x] A numerical value for number of cells or percentage (with statistics) is provided.

Methodology

Sample preparation	One million cells were stained in 100gL of Zombie Aqua (Fixable Viability Kit BioLegend; San Diego, California, USA) for twenty minutes at room temperature. Zombie aqua was removed and 20ul_ of antibody dilution was added to each sample, which was t en incubate on ice or 10-15 minutes. NODAL an CA9 staining was performed on fixed and permeabilized cells according to manufacturer instructions using the Fixation/Permeabilization kit (BD Biosciences).<br>Cells were washed with 200ul_ FACS buffer (PBS with 1% FBS), pelleted and then resuspended in 100ul 2% PFA in FACS buffer. For acquisition, cells were re-suspended in 300gl FACS buffer for flow acquisition. Doublet discrimination and live cell gates were used to identify the cells of interest, and quadrant gates were set according to the fluorescence minus one controls (FMO) (Supplemental Figure 6).
Instrument	BD FACSCanto II V96100493
Software	FACSDiva (BD Bioscience,) FLowJo (FLowJo L C)
Cell population abundance	Cancer stem cells as defined by CD44high/CD24low markers were a relatively rare population in our cell lines. Though they came to comprise nearly 50 of the population in some experiments.
Gating strategy	The initial gate was set on live cells using SSC-A and FSC-A. Doublet discrimination was completed using FSC-W and FSC-H, followed by SSC-W and SSC-H. Live cells were identified using Zombie Aqua (ZA) viability dye. Quadrant gates were set according to the fluorescence minus one controls (FMO) and cells in the top left quadrant were counted relative to the total number of events (10 000 ZA negative). See Supplemental Figure 6.

☒ Tick this box to confirm that a figure exemplifying the gating strategy is provided in the Supplementary Information.

Magnetic resonance imaging

Experimental design

Design type	*Indicate task or resting state; event-related or block design.*
Design specifications	*Specify the number of blocks, trials or experimental units per session and/or subject, and specify the length of each trial or block (if trials are blocked) and interval between trials.*
Behavioral performance measures	*State number and/or type of variables recorded (e.g. correct button press, response time) and what statistics were used to establish that the subjects were performing the task as expected (e.g. mean, range, and/or standard deviation across subjects).*

Acquisition

Imaging type(s)	*Specify: functional, structural, diffusion, perfusion.*
Field strength	*Specify in Tesla*
Sequence & imaging parameters	*Specify the pulse sequence type (gradient echo, spin echo, etc.), imaging type (EPI, spiral, etc.), field of view, matrix size, slice thickness, orientation and TE/TR/flip angle.*
Area of acquisition	*State whether a whole brain scan was used OR define the area of acquisition, describing how the region was determined.*

Diffusion MRI ☐ Used ☐ Not used

Preprocessing

Preprocessing software	*Provide detail on software version and revision number and on specific parameters (model/functions, brain extraction, segmentation, smoothing kernel size, etc.).*
Normalization	*If data were normalized/standardized, describe the approach(es): specify linear or non-linear and define image types used for transformation OR indicate that data were not normalized and explain rationale for lack of normalization.*
Normalization template	*Describe the template used for normalization/transformation, specifying subject space or group standardized space (e.g. original Talairach, MNI305, ICBM152) OR indicate that the data were not normalized.*
Noise and artifact removal	*Describe your procedure(s) for artifact and structured noise removal, specifying motion parameters, tissue signals and physiological signals (heart rate, respiration).*
Volume censoring	*Define your software and/or method and criteria for volume censoring, and state the extent of such censoring.*

Statistical modeling & inference

Model type and settings	*Specify type (mass univariate, multivariate, RSA, predictive, etc.) and describe essential details of the model at the first and second levels (e.g. fixed, random or mixed effects; drift or auto-correlation).*
Effect(s) tested	*Define precise effect in terms of the task or stimulus conditions instead of psychological concepts and indicate whether*

ANOVA or factorial designs were used.

Specify type of analysis: ☐ Whole brain ☐ ROI-based ☐ Both

Statistic type for inference
(See Eklund et al. 2016)

Specify voxel-wise or cluster-wise and report all relevant parameters for cluster-wise methods.

Correction

Describe the type of correction and how it is obtained for multiple comparisons (e.g. FWE, FDR, permutation or Monte Carlo).

Models & analysis

n/a	Involved in the study
☐	☐ Functional and/or effective connectivity
☐	☐ Graph analysis
☐	☐ Multivariate modeling or predictive analysis

Functional and/or effective connectivity

Report the measures of dependence used and the model details (e.g. Pearson correlation, partial correlation, mutual information).

Graph analysis

Report the dependent variable and connectivity measure, specifying weighted graph or binarized graph, subject- or group-level, and the global and/or node summaries used (e.g. clustering coefficient, efficiency, etc.).

Multivariate modeling and predictive analysis

Specify independent variables, features extraction and dimension reduction, model, training and evaluation metrics.
